# Soil moisture and microbiome explain greenhouse gas exchange in global peatlands

**DOI:** 10.1038/s41598-025-92891-z

**Published:** 2025-03-24

**Authors:** Jaan Pärn, Sandeep Thayamkottu, Maarja Öpik, Mohammad Bahram, Leho Tedersoo, Mikk Espenberg, John Alexander Davison, Kuno Kasak, Martin Maddison, Ülo Niinemets, Ivika Ostonen, Kaido Soosaar, Kristina Sohar, Martin Zobel, Ülo Mander

**Affiliations:** 1 Department of Geography, Institute of Ecology and Earth Sciences, University of Tartu, Tartu, Estonia; 2 Department of Botany, Institute of Ecology and Earth Sciences, University of Tartu, Tartu, Estonia; 3 Department of Ecology, Swedish University of Agricultural Sciences, Uppsala, Sweden; 4 Department of Agroecology, Aarhus University, Slagelse, Denmark; 5 Mycology and Microbiology Center, University of Tartu, Tartu, Estonia; 6 Department of Environmental Science, Policy, & Management, University of California, Berkeley, USA; 7 Institute of Agricultural and Environmental Sciences, Estonian University of Life Sciences, Tartu, Estonia

**Keywords:** Carbon cycle, Element cycles

## Abstract

Earth’s climate is tightly connected to carbon and nitrogen exchange between the atmosphere and ecosystems. Wet peatland ecosystems take up carbon dioxide in plants and accumulate organic carbon in soil but release methane. Man-made drainage releases carbon dioxide and nitrous oxide from peat soils. Carbon and nitrous gas exchange and their relationships with environmental conditions are poorly understood. Here, we show that open peatlands in both their wet and dry extremes are greenhouse gas sinks while peat carbon/nitrogen ratios are high and prokaryotic (bacterial and archaeal) abundances are low. Conversely, peatlands with moderate soil moisture levels emit carbon dioxide and nitrous oxide, while prokaryotic abundances are high. The results challenge the current assumption of a uniform effect of drainage on greenhouse gas emissions and show that the peat microbiome of greenhouse-gas sources differs fundamentally from sinks.

## Introduction

Future climates will be shaped by the balance between greenhouse gas sources and sinks in terrestrial ecosystems. Wet peatlands absorb annually 0.4 Pg of atmospheric carbon dioxide (CO_2_)^1^. All peatlands are the largest terrestrial carbon (C) stock (up to 535 Pg)^1^ and store one-tenth of all organic nitrogen (N)^2^. As a trade-off for the C storage^3^, wet peatlands are the largest natural source of methane (CH_4_), which is a 28 times more powerful greenhouse gas (GHG) than CO_2_ equivalent (CO_2_eq). N-rich drained peatlands also release nitrous oxide (N_2_O), the most dangerous destroyer of the stratospheric ozone layer and a GHG of 265 CO_2_eq. The balance between GHG fluxes in peatlands remains poorly understood, though there are known to be modulating effects of environmental factors, such as soil water content (SWC), soil temperature and availability of N^4^. SWC plays a key role in belowground ecosystems by determining the availability of water and oxygen for plant roots, fungi and prokaryotes (*i.e.* bacteria and archaea). Climate warming and drying, artificial drainage and land-use change have long-term implications for GHG exchange in peatlands^5–8^. Warming promotes metabolic rates, whereas SWC modulates warming-induced carbon fluxes^9^. Thus, a warmer climate aerates peat and promotes plant photosynthetic activity^10^ and fine-root growth, which releases more carbon to plant-associating fungi^11^. Moderate drying of wet soils enhances ecosystem respiration (ER)^12,13^ until it reaches a point of drought stress^10^. This produces a unimodal relationship of ER with soil moisture^9^. For net ecosystem exchange (NEE) of CO_2_, both negative^3,12^ and unimodal relationships^9,10,13,14^ with SWC have been proposed. N_2_O emissions from peatlands also peak at intermediate SWC, while wet and dry peatlands show negligible N_2_O emissions^4^. Net impact of drying on the balance between CO_2_ uptake vs. N_2_O and CH_4_ emission in peatlands is disputed^15–17^. However, the International Panel for Climate Change (IPCC) and other global surveys assert that CO_2_ represents the major component of global peatland GHG exchange, while CH_4_ and N_2_O play minor roles^2^. To understand GHG exchange in peatlands, we need to assess it in the context of C and N resource use and trade between plants and other biological kingdoms in peatland ecosystems. Plants photosynthesise organic C compounds including ‘easy’ ones, such as saccharides, and exude them into soil, which support bacteria and archaea. N fertilisation stimulates plants to allocate ‘easy’ C to root growth and root trait adjustment but reduces investment of C into mycorrhizal and other mutualisms, and leaves ‘easy’ C for bacteria and archaea^18–23^. Disturbances, most importantly land conversion, tillage, drainage or due to climate change, further promote dominance of non-mutualistic microbes^18–22^, primarily prokaryotes. Ecosystems on disturbed soils have low capacity to sequester and preserve C and N^18–23^ which is most integrally characterised by a low C/N ratio^24^.

**Fig. 1:**
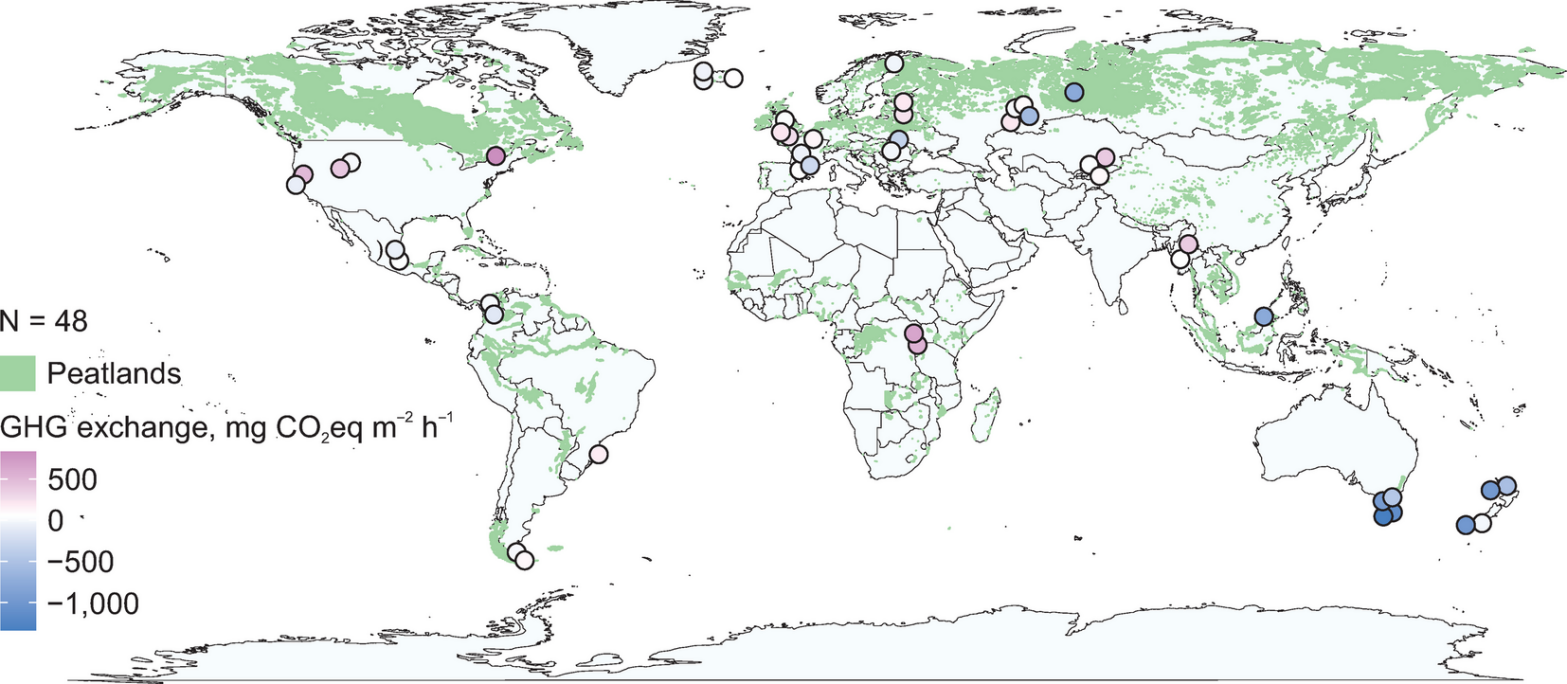
GHG exchange in open peatland study sites. Negative GHG exchange indicates net GHG uptake and positive GHG exchange indicates net GHG emission. Global peatland map: reference^8^.

Here, we analyse GHG exchange based on field chamber measurements of ER, N_2_O and CH_4_ fluxes^4^ and MODIS satellite data of gross primary production (GPP) in 48 open peatlands (Fig. 1) during the dry season. We further investigate explanatory factors of the GHG fluxes. We hypothesise that local environmental factors explain GHG exchange rates in the peatlands, while high gaseous C and N losses are associated with drained peat soils, low C/N ratios and high prokaryotic (bacterial and archaeal) abundances.

### Results and discussion

**Fig. 2:**
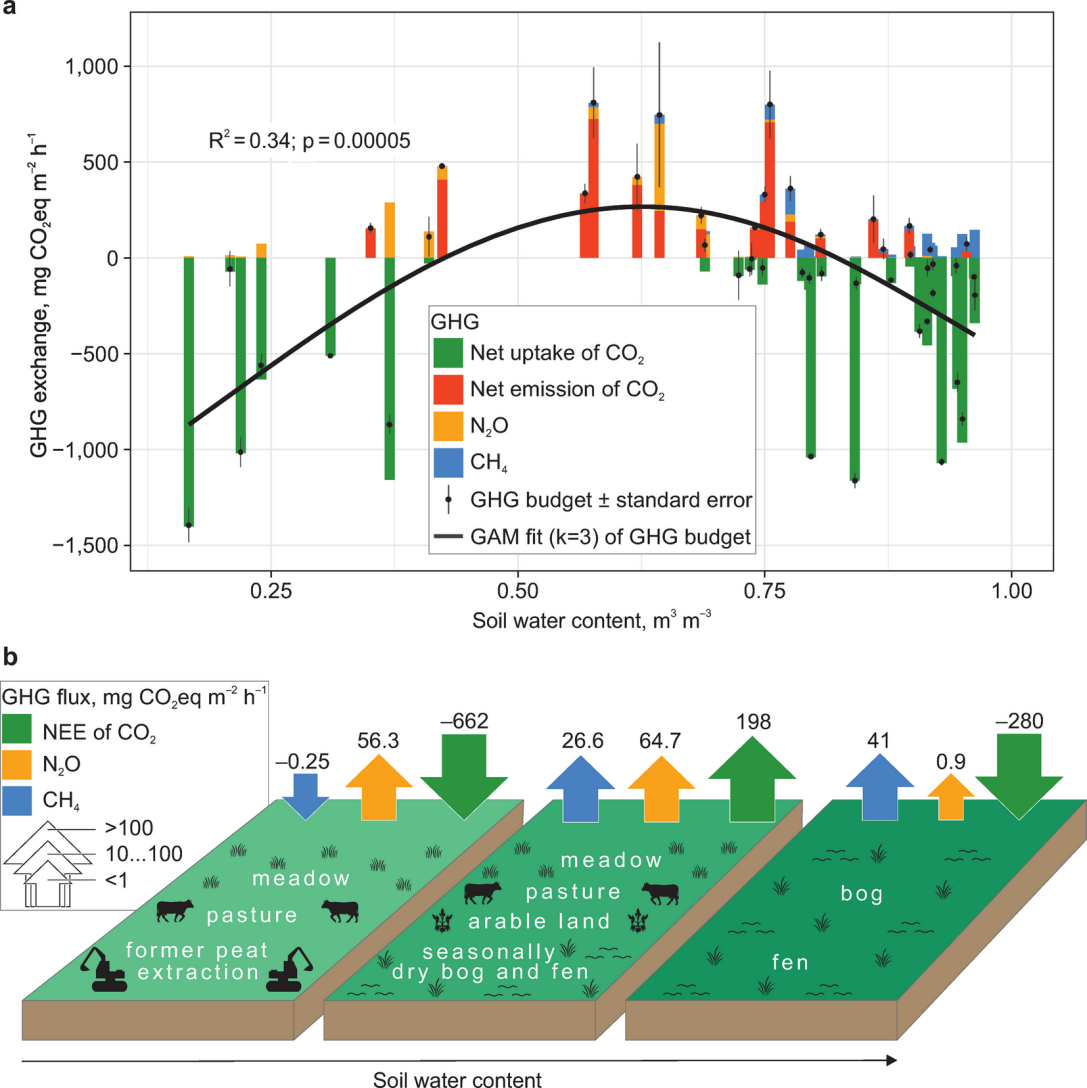
GHG exchange in peatland sites along the soil water content gradient. (**a**) breakdown of GHG budgets into individual fluxes; generalized additive model fit (k = 3) of GHG budgets as function of soil water content. Site average (bar or point) and standard error (whiskers) are shown. (**b**) GHG fluxes and land use along the soil water content gradient.

Our analysis showed that CO_2_ dominated GHG exchange in both net emitter and uptake sites (Fig. 2). In the latter, GPP clearly offset ER and CH_4_ emissions. In the high GHG source sites, the net emission of CO_2_ contributed > 83% of each GHG exchange. This corroborates the conclusion of the IPCC and several other global studies that CO_2_ is the predominant GHG, while CH_4_ is a minor component^2^. N_2_O contributed > 33% of each GHG exchange value in four GHG source sites (drained floodplain meadows and cultivated fields) and > 10% in other drained grasslands. This is consistent with the previous notion that N_2_O emissions are mostly confined to restricted locations and events (i.e. hot spots and hot moments). The GHG-neutral sites (between –100 and +100 mg CO_2_eq m^–2^ h^–1^) experienced modest fluxes of all three GHGs (Fig. 2a).

NEE and total GHG exchange values were both unimodally distributed along the dimension of soil water content (generalized additive model (GAM) R^2^_adj_ = 0.31 and 0.34 for NEE and GHG exchange, respectively; Fig. 2a; Extended Data Fig. 1): while moderately moist sites were CO_2_ and GHG sources, both wet and dry peatlands were net CO_2_ and total GHG sinks. The upward slope between the wet and moderately moist peatlands corresponds to the well-known response of ER to drainage, which, without a matching increase in GPP, promotes NEE^3,12^. However, the decline from moderately moist towards dry peat questions the current assumption of a universal positive effect of drainage on CO_2_ and GHG emissions^3,12^. Instead, the drop in NEE appears to indicate drought stress on heterotrophic respiration^10^ in the dry grasslands, while high GPP is maintained. The form of the relationship is consistent with previous unimodal CO_2_ curves in peatlands^9,10,13,14^. Here, we show that a global optimum of CO_2_ and GHG exchange is observed at ~ 0.6 m^3^ m^–3^ SWC within the full soil moisture spectrum of peatlands.

**Fig. 3:**
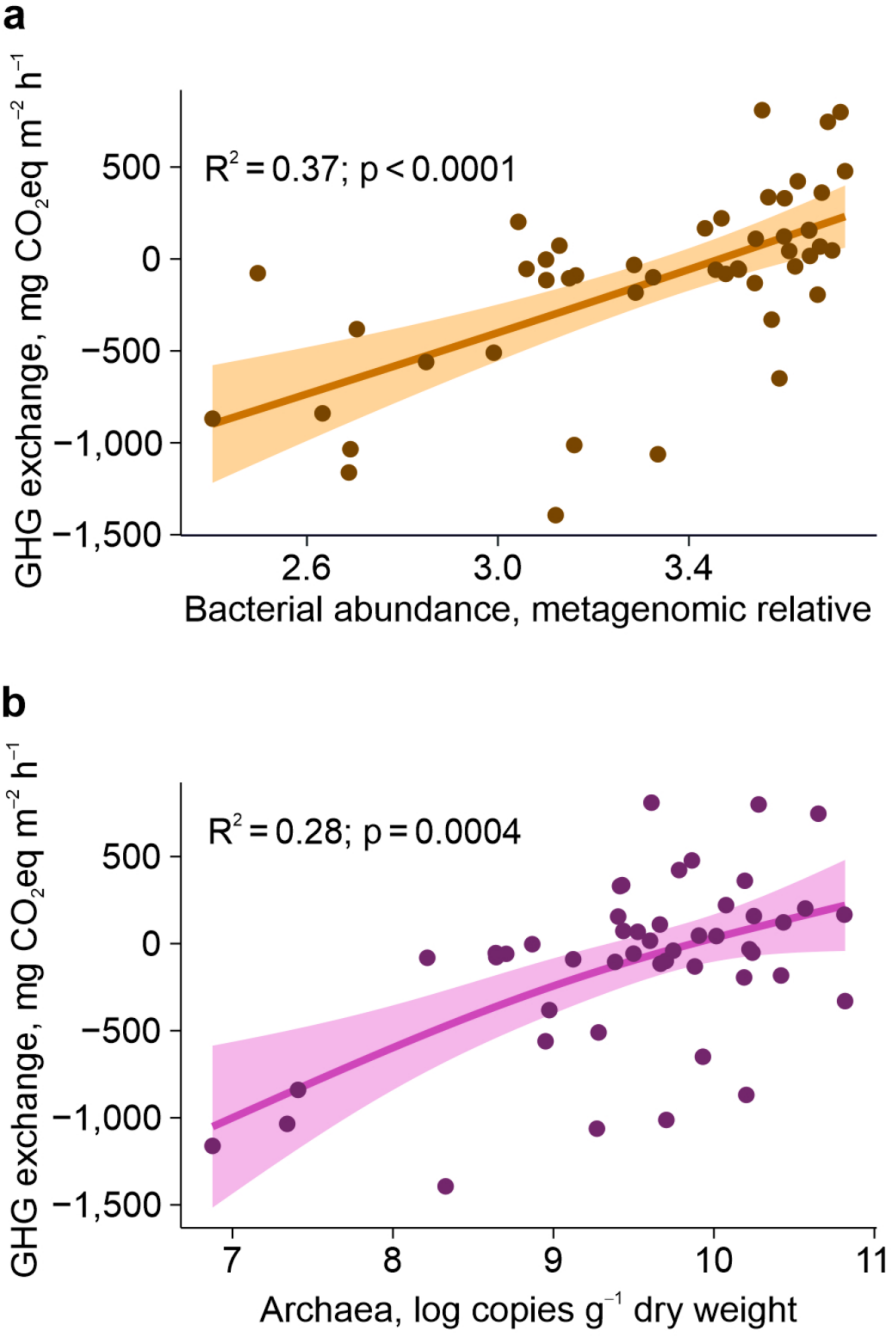
GHG exchange in relationship with soil prokaryotic abundances. (**a**) GHG exchange vs. sum of metagenomic relative abundance of soil bacteria; (**b**) GHG exchange vs. qPCR-measured gene copy numbers of archaea per gram of dry soil (copies g^–1^ dry weight). Negative GHG exchange shows net uptake in plants and positive GHG exchange shows net emission on top of the uptake.

The relative bacterial and archaeal abundances of soil explained another good part of variation of GHG exchange rates across the peatlands (GAM R^2^_adj_ = 0.37 and 0.28 between GHG emission, and archaea or bacteria, respectively; Fig. 3) while no fungal guild showed a fair correlation with the GHG. Low C/N ratio was the main abiotic factor behind the high bacterial and archaeal abundances (linear R^2^_adj_ = 0.41 and 0.28, respectively). No bacterial or archaeal phylum showed strong correlation to GHG exchange, as only *Nitrospirae, Parcubacteria, Deltaproteobacteria* and *Bathyarchaeota*, known for wide metabolic capabilities, moderately correlated with GHG emissions (0.20 < R^2^_adj_ < 0.30). Multiple-regression GAM models involving SWC and soil prokaryotic abundances predicted the GHG exchange rates well (R^2^_adj_ = 0.57 with bacteria and R^2^_adj_ = 0.60 with archaea). The findings refine the idea that high carbon emissions from ecosystems experiencing disturbance, N fertilisation (including peat mineralisation), and low C and N preservation capacity^18,20–22^ are linked to a high proportion of microbial generalists^18,19,22^. We initially suspected mycorrhizal fungi behind the relationships. However, neither mycorrhizal fungal abundance nor its ratio to prokaryotes was correlated with GHG exchange. Instead, we suppose the mechanism is competition between plant-associated mutualisms and non-mutualistic microbes^18,19,21,22^. C and N sources are better available for prokaryotes in poorer plant–fungi collaboration, wherefore higher prokaryotic abundances may indicate habitats where prokaryotes flourish while plant–fungi mutualisms fail^6,7,11,12,22,23^.

CH_4_ emissions were best explained by log-linear relationships with SWC (R^2^_adj_ = 0.38) and water table height (R^2^_adj_ = 0.34; Extended Data Fig. 2). Thus, dry peats (< 0.5 m^3^ m^–3^ SWC) took up or emitted only small amounts of CH_4_ (< 0.1 mg C m^–2^ h^–1^) whereas all high CH_4_ emissions were produced in water-saturated peat. This was expected from the strictly anaerobic process of methanogenesis. Furthermore, peatlands showed a tendency towards higher emissions > 13 °C soil temperatures (20 cm depth: R^2^_adj_ = 0.06; p = 0.047, Extended Data Fig. 3). The modest fit of the environmental CH_4_ models can be explained by the intrinsic confinement of CH_4_ emissions to individual emission hot spots. However, as CH_4_ is a minor component of GHG exchange (Fig. 2), the > 60% uncertainty in CH_4_ flux estimates does not translate into large uncertainty in GHG exchange across global peatlands. N_2_O emissions were log–log linearly related to soil nitrate content and formed a unimodal relationship with SWC^20^.

Here are some limitations of this study. The study is based on field chamber measurements and MODIS satellite data during the dry season, which may not fully capture the spatial and temporal variability of GHG fluxes across different seasons and weather conditions. However, previous studies have shown that the annual minimum water table is an integral characteristic of annual GHG fluxes in peatlands^25–27^. The MODIS GPP product has been independently validated against chamber measurements in open peatlands with excellent matches^28,29^. Overestimation of MODIS GPP in dry grasslands has been suspected, owing to a proposed > 50% underestimate of a negative effect of drought^30^. On an opposite note, validation with flux towers has shown underestimation of MODIS GPP^31^. As another caveat, *e*_*max*_ Eq. (2) of MODIS GPP depends heavily on land cover type. Open peatlands are not a land cover type on its own but are distributed between wetlands, grasslands and croplands. The underestimation is low for croplands and grasslands^31^, and is mostly the problem in forests, which we did not analyse here. A multi-scale analysis has shown the accuracy of MODIS GPP product depends on calibration methods, with flux towers generally showing larger GPP than chambers^32^. The same analysis showed that the MODIS GPP product is robust at all scales, including at different microtopographic sites^32^. Duration-wise, we assumed the vegetation in the 8-day MODIS GPP product window as representative of our sampling dates, which has been shown as producing negligible error^33^. Accordingly, we extracted GPP values for our sites from the dataset (kg C m^–2^ 8 days^–1^) specifically for our field visit dates. Therefore, this study acknowledges a significant uncertainty in GHG flux estimates, which can exceed 60%. This uncertainty could affect the overall accuracy of GHG exchange measurements. We tested the significance of the possible overestimation^30^ in dry sites by multiplying the GHG exchange values from our dry (< 0.4 m^3^ m^–3^ SWC) by a factor of 0.5 and using them in the regression analyses. The patterns of GHG exchange values vs. SWC (Fig. 2; Extended Data Fig. 1) after this reduction became less pronounced but retained their significance. Thus, the main patterns we observed still hold after the test. As a further limitation, our findings are based on 48 open peatland sites under a broad range of land-use regimes, which may still not be representative of all peatland ecosystems globally. The results might not be directly applicable to peatlands with different environmental conditions or management practices. The limitations highlight the need for further research to improve the understanding of GHG exchange in peatlands, considering a broad range of environmental factors and comprehensive microbial analyses.

Taken together, future impacts of global change on GHG exchange and the state of peatland ecosystems will be determined by drying and mineralisation of peat. GHG sequestration potential of undisturbed wet peatlands and emissions from moderately drained peat are relatively well published. Conservation is by far the most efficient management strategy for natural peatlands. Wetlands with low soil prokaryotic abundances should also be a conservation priority for their inherently well-developed carbon and nitrogen sequestration capacity. Our findings of GHG sinks in dry peatlands support recultivation with drought-tolerant vegetation and maintenance of an artificially low water table for those drained peatlands that cannot be feasibly restored as wetlands. Peatlands with high soil prokaryotic abundances present the greatest potential for revegetation to improve carbon preservation capacity. Time will need to be allowed to move beyond the short-term carbon and nitrogen management policies for peatlands.

## Methods

### Field sampling

We conducted a survey of CO_2_, CH_4_ and N_2_O fluxes and potentially controlling environmental variables at peatland sites globally, during the dry season (i.e. the annual water table minimum time of year including temperate and boreal summers) at each site between 2011 and 2018. We selected a total of 48 open (i.e., with vegetation height < 0.5 m inside and around the chambers) peatland sites (Fig. 1) from our global wetland soil database^4,34^ throughout the rainy tropical (A), temperate (C), and boreal (D) climate zones of the Köppen classification (Fig. 1). We identified natural and artificially drained sites based on the proximity of drainage ditches, water table height, and characteristic vegetation. The hydrology and trophic status of the natural sites ranged from groundwater-fed swamps and fens to rain-fed peat bogs. We also selected the sites to represent the full typical range of land uses of each study region. Accordingly, our study sites represent peatlands that have been arable lands for > 5 years (Borneo, Myanmar, Tasmania and Uganda), abandoned peat extraction areas (Russia and Tasmania), intensively (more than once a year) grazed or mown peatlands (Brazil, Colombia, Estonia, Kyrgyzstan, New Zealand, Quebec, Tasmania and Uganda), non-intensively (once a year) grazed or mown peatlands (California, Catalonia, Estonia, France, Iceland, Kyrgyzstan, Mexico, Montana, Myanmar, Russia, New Zealand and Wales) and a peatland under no human land use in each study region, hence distributed uniformly across the world’s peatlands. To capture the full variety of GHG fluxes at a site, we set up transects of 2–3 points per transect, each point containing 3–4 opaque truncated conical chambers, arranged along 25–100 m of terrain. Gas concentrations were sampled during 3–6-day campaigns using the static chamber method with PVC collars of 0.5 m diameter and 0.1 m depth installed in the soil^4,28^. The gas samples were collected into pre-evacuated 50 mL glass vials between 8 am and 8 pm to represent the average diurnal emissions^35^. The gas samples were transported to our laboratory at the University of Tartu and analysed by gas chromatography (GC-2014; Shimadzu, Kyōto, Japan) instrumented with an electron capture detector for detection of N_2_O and a flame ionisation detector for CH_4_, and Loftfield-type autosamplers. We calculated ER, CH_4_ and N_2_O fluxes (in mg m^–2^ h^–1^) using changes in concentration during one hour within the chamber. Accordingly, gas concentration was measured at 20 min intervals (0, 20, 40 and 60 min). An individual gas flux was determined from the linear regression obtained from the consecutive concentrations. We closely examined the shape of our gas concentration trends in each individual chambers. Practically all significant deviations from a linear trend were apparently caused by a faulty chamber sealing. We did not observe any signs of ebullition such as jump rises in concentration not followed by a drop in concentration. An only small share of ebullition may be a peculiarity of our long chamber closing time of 1 h. A p level of < 0.05 was accepted for the goodness of fit to linear regression. Insignificant fluxes (p > 0.05) below the accuracy of gas chromatograph (regression change of gas concentration δv < 10 ppb) were included in the analysis as zeros.

Each transect point was instrumented with a 1-m-deep observation well (a 50-mm-diametre perforated PP-HT pipe wrapped in geotextile). Water-table height was recorded daily from the observation wells during the gas sampling. Soil temperature was measured at 10, 20, 30, and 40 cm depths. We collected soil samples of 150–200 g from the chambers at 0–10 cm depth after the final gas sampling, and transported them to laboratories in Tartu, Estonia.

### Estimation of GPP

As the estimate of GPP, we used MOD17A2H 8-day 500 m grid V006 data^36^ developed from the MODIS sensor data onboard the Terra and Aqua satellites and expressed in kg CO_2_ m^–2^ 8 days^–1^. MOD17A2H V006 is based on the radiation use efficiency concept^37^ with three major components. The first assumption is that GPP is directly related to the solar energy absorbed by plants. Second, the concept assumes a connection between absorbed solar energy and satellite-derived spectral indices such as NDVI. The third assumption is that for biophysical reasons, the actual conversion efficiency of absorbed solar energy is lower than the theoretical value. The calculation of GPP Eq. (1) requires radiation use efficiency and absorbed photosynthetically active radiation (APAR) measurements. APAR calculates the available leaf area index (LAI) to absorb incident solar energy. This estimate is then converted into GPP by multiplying APAR with radiation use efficiency (e) Eq. (1). Remote sensing data usually provide the fraction of photosynthetically active radiation (FPAR). APAR can be calculated by Eq. (4)^38^. This requires the estimation of incidental photosynthetically active radiation (IPAR) Eq. (5), which is extracted from the GMAO/NASA dataset^36^.

1 $$GPP \, = e*APAR$$

2 $$e = e_{max} *T_{min\_scalar} *VPD\_scalar$$

3 $$FPAR = APAR/PAR \approx NDVI$$

4 $$APAR \, = \, IPAR*FPAR$$

5 $$IPAR \, = \, SWR_{rad} *0.45$$

*e*_*max*_ is the maximum radiation conversion efficiency in kg C MJ^–1^ which is obtained from the Biome Properties Look-Up Table (BPLUT) of the at-launch land cover product of MODIS (MOD12)^32^.

*T*_*min_scalar*_ and *VPD_scalar* are the ramp functions of *T*_min_ and VPD. This calculation requires the following parameters extractable from the GMAO/NASA dataset^38^.

*T*_*min_max*_ (ºC)—the daily minimum temperature at which e = e_max_ for an optimal VPD.

*T*_*min_min*_ (ºC)—the daily minimum temperature at which e = 0 at any VPD.

*VPD*_*max*_ (Pa)—the daylight average vapor pressure deficit at which e = e_max_ for an optimal T_min_.

*VPD*_*max*_ (Pa)—the daylight average vapor pressure deficit at which e = 0.0 at any T_min_.

SWR_rad_ = Incident shortwave radiation used for calculating IPAR.The values were converted to mg C m^–2^ h^–1^ as follows:

6 $${\text{GPP}} = {\text{MODIS GPP}} \cdot {1},000,000/\left( {{\text{8 days}} \cdot {\text{24 h}}} \right)$$

where GPP was gross primary production transformed to mg C m^–2^ h^–1^.

We calculated NEE from GPP and ER as follows:

7 $$NEE = ER{-}GPP$$

GHG exchange was calculated for each chamber as follows.

8 $$GHG \, exchange = CH_{4} \cdot GWP_{CH4} + N_{2} O \cdot GWP_{N2O} + NEE{\text{where}}:$$

*GHG exchange* was the greenhouse gas exchange in CO_2_ equivalents (CO_2_eq),

*CH*_*4*_ was the field-observed methane flux, mg CH_4_ m^–2^ h^–1^,

*GWP*_*CH4*_ was 28 CO_2_eq, the 100-year global warming potential of CH_4_ without climate–carbon feedbacks according to the IPCC Fifth Assessment Report,

*N*_*2*_*O* was the field-observed nitrous oxide flux, mg N_2_O m^–2^ h^–1^,

*GWP*_*N2O*_ was 265 CO_2_eq, the 100-year global warming potential of N_2_O without climate–carbon feedbacks according to the IPCC Fifth Assessment Report, and.

NEE was the net ecosystem exchange of CO_2_ (Eq. 6).

### Laboratory inorganic chemical and soil physical analyses

The homogenised samples were divided into subsamples for physical–chemical analyses and DNA extraction. Plant-available phosphorus (P, NH_4_-lactate extractable) was determined on a FiaStar5000 flow-injection analyser^40^. Plant-available potassium (K) was determined from the same solution by the flame-photometric method and plant-available magnesium (Mg) was determined from a 100 mL NH_4_-acetate solution with a titanium-yellow reagent on the flow-injection analyser^40^. Plant-available calcium (Ca) was analysed using the same solution by a flame-photometrical method. Soil pH was determined using a 1N KCl solution; soil NH_4_ and NO_3_ were determined on a 2 M KCl extract of soil by flow-injection analysis (APHA-AWWA-WEF). Total N and C contents of oven-dry samples were determined by a dry-combustion method on a varioMAX CNS elemental analyser (Elementar Analysensysteme GmbH, Germany). Organic matter content of dry matter was determined by loss on ignition^40^. We determined SWC from gravimetric water content (GWC), dry matter content and empirically established bulk densities (BD) of mineral and organic matter fractions^4,41–43^ as follows:

9 $${\text{SWC}} = {\text{GWC}} \cdot {\text{BD}}$$

where: SWC is soil water content, m^3^ m^−3^,

GWC is gravimetric water content, Mg Mg^−1^, calculated as the difference between the fresh and oven-dry weight divided by the oven-dry weight^41^, and

BD is bulk density, Mg m^−3^.

### DNA extraction, quantitative PCR and metagenomics

DNA extraction was performed from 0.2 g of frozen soil samples using the Qiagen Dneasy PowerSoil Kit (12888-100), following the manufacturer’s recommendations. DNA concentrations were measured with the Qubit™ 1X dsDNA HS Assay Kit using a Qubit 3 fluorometer (Invitrogen)^34^.

For soil archaeal and bacterial abundances, real-time quantitative polymerase chain reaction (qPCR) assays were performed using a RotorGene® Q equipment (Qiagen, Valencia, CA, USA). Amplification was carried out in 10 μL reaction solutions containing 5 μL Maxima SYBR Green Master Mix (Thermo Fisher Scientific Inc., Waltham, MA, USA), with an optimised concentration of forward and reverse primers, 1 μL of template DNA and sterile distilled water. qPCR measurements were performed in triplicate, and the absence of contaminations was verified against negative controls. For a detailed description of the gene-specific primer sets, thermal cycling conditions, and primer concentrations for the bacterial and archaeal 16S rRNA, see^44^. Quantitative data were analysed with RotorGene Series Software v. 2.0.2 (Qiagen) and the LinRegPCR program v. 2018.0^45^. The archaeal and bacterial gene abundances were calculated as mean fold differences between samples and corresponding tenfold standard dilution in respective standards, as recommended by^45^; the gene abundances were reported as gene copy numbers per gram of dry soil (copies g^–1^ dry weight).

We calculated metagenomic relative abundances (*i.e.* miTag^46^) of archaea, bacteria and fungi based on small subunit (SSU) rRNA genes^34^. For this, SortMeRNA (version 2.0)^47^ was used to extract and blast search rRNA genes against the SILVA SSU database (v128). Reads approximately matching this database with e < 10^−4^ were further filtered with custom Perl and C +  + scripts and merged using FLASH. In case read pairs could not be merged, the reads were interleaved such that the second read pair was reverse complemented and then sequentially added to the first read. Of these preselected reads, 50,000 reads were fine-matched the Silva SSU database using Lambda and the lowest common ancestor (LCA) algorithm adapted from LotuS.

For calculating relative abundance of different fungal guilds, we performed metabarcoding using PacBio sequencing. The sequencing data were analysed using the PipeCraft pipeline^48^. We used the FungalTraits database^49^ for functional annotation of the data. We calculated metagenomic relative abundances of fungi based on small subunit (SSU) rRNA genes, blasted against the Silva SSU database. The read abundance was normalised by the total number of metagenomic SSU reads.

### Correlation analysis of GHG against environmental factors and soil microbiome characteristics

We calculated a correlation matrix between our individual GHG fluxes and their total CO_2_eq exchange values, environmental factors, relative abundances of functional groups of microbes and ratios between them. We used linear and non-parametric GAM models applying minimal smoothness (k = 3)^4^. We assessed the normality of our data using visual approaches and the Shapiro–Wilk test. Where necessary, we log-transformed the values. For the GHG flux rates, we considered the following environmental predictor variables: soil and water temperature, distance from the equator, Köppen climate zone (A, C or D), water table, volumetric SWC, soil chemistry (pH, total C%, organic matter, total N%, C:N ratio, ammonium, nitrate, calcium, magnesium, potassium and phosphorus), water oxygen content, and agricultural land use intensity. We calculated the correlation matrix using the R programming language (*stats* and *mgcv* packages). We reported correlations with a significance level of p = 0.05.

## Supplementary Information

Supplementary Information 1.

Supplementary Information 2.

Supplementary Information 3.

Supplementary Information 4.

Supplementary Information 5.

Supplementary Information 6.

Supplementary Information 7.

## Data Availability

The study is mostly based on data published in^4,34^. Additional source data (Figs. 1–3) are provided with this paper.
