# Supplementary figures and images for: Soil moisture and microbiome explain greenhouse gas exchange in global peatlands
Source: Sci Rep. 2025 Mar 24;15:10153. doi: 10.1038/s41598-025-92891-z (PMC11933456; doi:10.1038/s41598-025-92891-z)

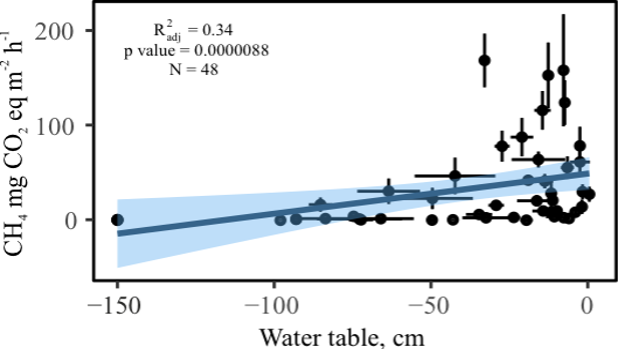

Supplement: Supplementary file 1 — Supplementary Information 1. [file 41598_2025_92891_MOESM1_ESM.png]

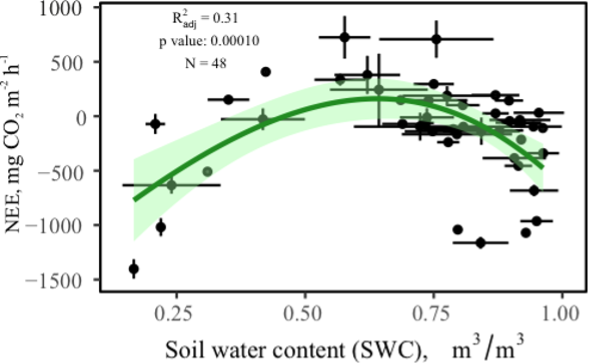

Supplement: Supplementary file 2 — Supplementary Information 2. [file 41598_2025_92891_MOESM2_ESM.png]

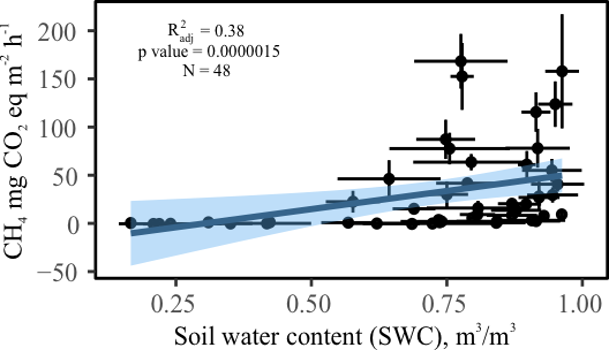

Supplement: Supplementary file 3 — Supplementary Information 3. [file 41598_2025_92891_MOESM3_ESM.png]

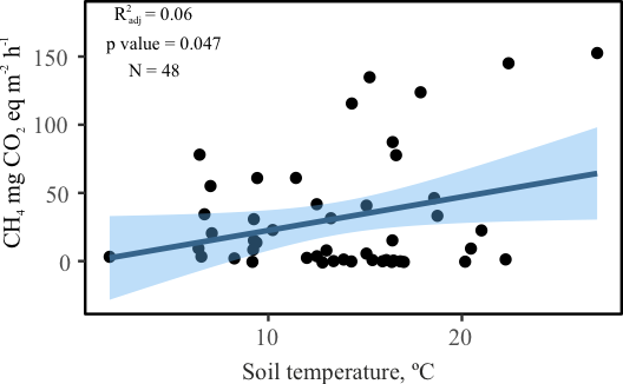

Supplement: Supplementary file 4 — Supplementary Information 4. [file 41598_2025_92891_MOESM4_ESM.png]
